# Oral Delivery of Novel Recombinant *Lactobacillus* Elicit High Protection against *Staphylococcus aureus* Pulmonary and Skin Infections

**DOI:** 10.3390/vaccines9090984

**Published:** 2021-09-03

**Authors:** Na Pan, Bohui Liu, Xuemei Bao, Haochi Zhang, Shouxin Sheng, Yanchen Liang, Haiting Pan, Xiao Wang

**Affiliations:** 1 State Key Laboratory of Reproductive Regulation and Breeding of Grassland Livestock, Inner Mongolia University, Hohhot 010070, China; 22008032@mail.imu.edu.cn (N.P.); 31808136@mail.imu.edu.cn (B.L.); 31908096@mail.imu.edu.cn (X.B.); 21908028@mail.imu.edu.cn (H.Z.); shouxins1230@mail.imu.edu.cn (S.S.); yanchenliang@mail.imu.edu.cn (Y.L.); 131985999@imu.edu.cn (H.P.); 2 Basic Medical College, Inner Mongolia Medical University, Hohhot 010110, China

**Keywords:** *Staphylococcus aureus*, oral vaccine, *Lactobacillus*, mucosal delivery

## Abstract

*Staphylococcus aureus* is a leading cause of nosocomial and community-associated infection worldwide; however, there is no licensed vaccine available. *S. aureus* initiates infection via the mucosa; therefore, a mucosal vaccine is likely to be a promising approach against *S. aureus* infection. Lactobacilli, a non-pathogenic bacterium, has gained increasing interest as a mucosal delivery vehicle. Hence, we attempted to develop an oral *S. aureus* vaccine based on lactobacilli to cushion the stress of drug resistance and vaccine needs. In this study, we designed, constructed, and evaluated recombinant *Lactobacillus* strains synthesizing *S. aureus* nontoxic mutated α-hemolysins (Hla_H35L_). The results from animal clinical trials showed that recombinant *Lactobacillus* can persist for at least 72 h and can stably express heterologous protein in vivo. Recombinant *L. plantarum* WXD234 (pNZ8148-Hla) could induce robust mucosal immunity in the GALT, as evidenced by a significant increase in IgA and IL-17 production and the strong proliferation of T-lymphocytes derived from Peyer’s patches. WXD234 (pNZ8148-Hla) conferred up to 83% protection against *S. aureus* pulmonary infection and significantly reduced the abscess size in a *S. aureus* skin infection model. Of particular interest is the sharp reduction of the protective effect offered by WXD234 (pNZ8148-Hla) vaccination in γδ T cell-deficient or IL-17-deficient mice. In conclusion, for the first time, genetically engineered *Lactobacillus* WXD234 (pNZ8148-Hla) as an oral vaccine induced superior mucosal immunity, which was associated with high protection against pulmonary and skin infections caused by *S. aureus*. Taken together, our findings suggest the great potential for a delivery system based on lactobacilli and provide experimental data for the development of mucosal vaccines for *S. aureus*.

## 1. Introduction

*Staphylococcus aureus* is normally a non-harmful commensal bacterium in humans and other mammals, but occasionally becomes invasive and produces serious diseases, including sepsis, infective endocarditis, pleurisy, septic arthritis, skin infection, and soft tissue infection [1]. Routine, indiscriminate use of antibiotics during recent decades has resulted in the increasing development of drug-resistant strains, especially methicillin-resistant *S. aureus* (MRSA) and vancomycin-resistant *S. aureus* (VRSA), characterized by wide spreading ability, high pathogenicity, multidrug resistance, and limited or no options for therapeutic intervention [2,3]. Morbidity and mortality due to serious diseases and their sequelae are high despite use of antibiotics to which these strains are susceptible [4]. Vaccination is a promising method, in theory, for preventing and combating *S. aureus* infections without risking the development of drug-resistant strains [5]. However, notwithstanding several decades of intensive research by numerous first-class research institutions, a safe and consistently effective vaccine against *S. aureus* is still not available—and is urgently needed [6,7,8].

Host mucosal tissues such as gut and skin are cohabitated. *S. aureus* normally colonizes mucous membranes and skin in a non-harmful manner but can quickly take advantage of opportunities to invade adjoining tissues if these barriers are overcome, in which case, mucosal immune response is an important first line of defense. The delivery of a vaccine directly to mucosa is therefore likely to help stimulate protective immunity at the invasion site. Oral vaccines can stimulate protective immunity at the different invasion site, for instance, the Oral Polio Vaccine^®^ [9] and Dukoral^®^ [10] take action in the intestine and oral influenza vaccines [11,12] and *Streptococcus pneumoniae* vaccines are active in the respiratory tract [13]. Studies to date on anti-*S. aureus* mucosal vaccines have been restricted to those based on live bacteria [14], *S. aureus* antigens [15,16], nanoparticles [17], or bacterial outer membrane vehicles [18]. The effectiveness and safety of such vaccines remain to be confirmed.

*Lactobacillus* strains are important members of the human and animal microbiome and have gained wide-spread attention because of their diverse effects on host health. Several features of lactobacilli make them strong candidates as vectors for oral delivery to the mucosa of compounds of pharmaceutical interest, particularly vaccines and immunomodulators. Lactobacilli have received the “generally recognized as safe” (GRAS) designation from the US FDA (GRAS notice website: https://www.accessdata.fda.gov/scripts/fdcc/?set=GRASNotices (accessed on 14 June 2021)) and have been widely utilized in food and pharmaceutical industries for decades. Strains of the genus *Lactobacillus* are resistant to toxic effects of bile acid and are persistent in the gastrointestinal tract [19]. Prototypes of modified *Lactobacillus* strains are being developed, and recombinant forms of various *Lactobacillus* species have been used as a basis for vaccines against *Streptococcus pneumoniae* [20], transmissible gastroenteritis coronavirus [21], *Bacillus anthracis* [22,23], rotavirus [24], and tetanus toxin [25]. The possible utilization of lactobacilli as a delivery system for a *S. aureus* antigen has not been investigated. Hence, we here describe a promising strategy along this line for the generation of an effective *S. aureus* oral vaccine utilizing lactobacilli as a delivery vector.

Previously, two lactobacilli possessing tremendous probiotic properties were isolated from dairy products and were identified as *L. kefiri* SXJ29 (GenBank: MZ501823.1) and *L. plantarum* WXD234 (GenBank: MZ501859.1) [26]. In this study, we described new anti-*S. aureus* oral vaccines based on Hla_H35L_ [27,28]-expressing lactobacilli strains. The efficacy of recombinant *Lactobacillus* WXD234 (pNZ8148-Hla) was comprehensively evaluated using two mouse models suitable for accurately monitoring the progression of several diseases, including pulmonary and skin infections. These results suggest a great potential for a delivery system based on lactobacilli and provide experimental data for the development of *S. aureus* mucosal vaccines.

## 2. Materials and Methods

### 2.1. Bacterial Strains and Growth Conditions

The detail of all strains and plasmids used in this study are listed in Table 1. *Lactobacillus* strains were grown in de Man, Rogosa and Sharpe (MRS) (HuanKai Microbial; Guangdong, China) broth at 37 °C in anaerobic conditions for 24 h. The *Lactobacillus* derivatives were cultured with the addition of chloramphenicol (20 μg/mL; antibiotic) and nisin (5 ng/mL; inducer). *S. aureus* USA300 was cultured in Luria–Bertani (LB) medium at 37 °C in aerobic conditions for 16 h.

### 2.2. Experimental Animals and Ethics Statement

Specific-pathogen-free (SPF) female wild-type (WT) C57BL/6 mice were purchased from the Beijing Vital River Laboratory Animal Technology Co. (Beijing, China). TCR γ/δ-deficient mice (JAX Stock number: 003288) and IL-17A-deficient mice (NCBI Stock number: 16171) of C57BL/6N background were kindly donated by Dr. Z. Yin (College of Life Sciences, Jinan University, Guangdong, China). All of the animal-related experimental protocols applied in this study were conducted under the standards of the Ethics Committee of Inner Mongolia Medical University (SCXK2016-0001).

### 2.3. Construction of Hla_H35L_ Cell Surface Display Lactobacillus Based on SXJ29 and WXD234

**Figure 1:**
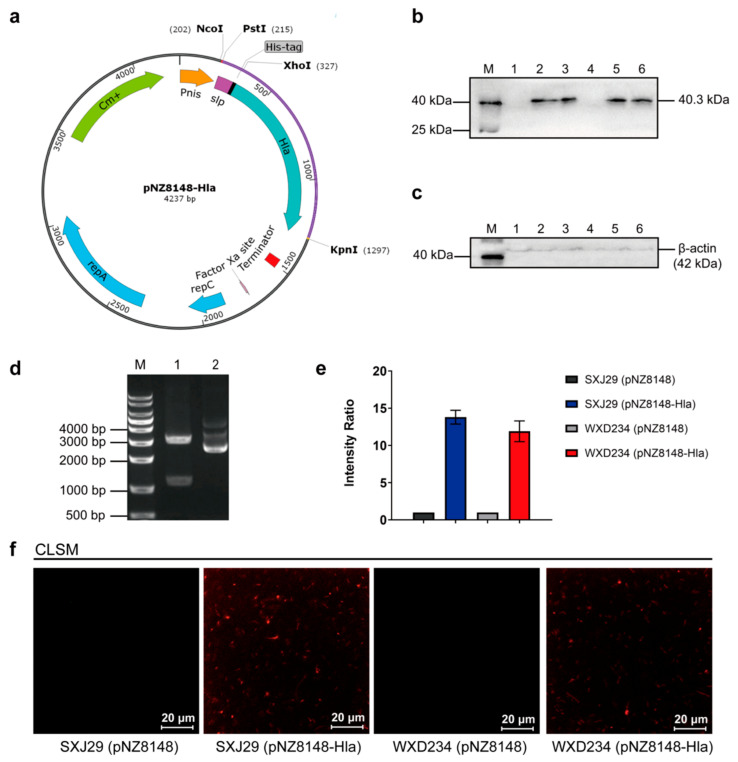
Construction of the pNZ8148-Hla plasmid and the expression of Hla_H35L_ in SXJ29 and WXD234. (**a**) Genetic engineering using pNZ8148 (schematic). (**d**) Full-length coding sequence of Hla_H35L_ was cloned into the pNZ8148 plasmid and was modified with a signal peptide from *L. brevis* and a His-tag. The recombinant pNZ8148-Hla plasmid was identified by restriction analysis. DNA markers (Lane M), pNZ8148-Hla (Lane 2), and pNZ8148-Hla were digested with PstI and KpnI (lane 1). Hla_H35L_ expression on recombinant SXJ29/WXD234 (pNZ8148-Hla) subjected to nisin induction was measured by Western blotting analysis. Cell extracts were prepared and analyzed by means of Western blotting using anti-Hla_H35L_ mAb (**b**) or anti-β-actin mAb (**c**). Left: molecular masses of pre-stained marker proteins. Protein markers (lane M), SXJ29 (pNZ8148) (Lane 1), SXJ29 (pNZ8148-Hla) (Lane 2, 3), WXD234 (pNZ8148) (Lane 4), WXD234 (pNZ8148-Hla) (Lane 5, 6). (**e**) Intensity ratio of Hla_H35L_ expression in SXJ29 and WXD234. (**f**) CLSM detection of Hla_H35L_ expression on SXJ29/WXD234 (pNZ8148-Hla).

The design strategy and construction process of the recombinant plasmid pNZ8148-Hla are shown in Figure 1a. A 90-bp fragment encoding *L. brevis* S-layer 30-a.a. signal peptide [29] was ligated to the front of the *S. aureus* antigen Hla_H35L_. The *hla_H35L_* gene was designed based on the *L. plantarum* codon bias and was artificially synthesized by GenScript Biotech (Nanjing, China). The fragment was ligated into the *EcoR*V restriction site in plasmid pUC57 to generate the cloning plasmid pUC57-Hla. The *sp-hla_H35L_* fragment obtained by the digestion of pUC57-Hla with *Nco*I and *Kpn*I was inserted into the *Lactococcus lactis* inducible expression plasmid pNZ8148 to generate the recombinant expression plasmid pNZ8148-Hla. Competent *Lactobacillus* were prepared through the inoculation (1:25) of SXJ29 overnight culture in MRS containing 1% glycine and of WXD234 in MRS containing 0.3 M sucrose. SXJ29 bacteria were cultured until the OD_600_ value was from 0.6–0.8 and were collected by centrifugation and washed 2× with 10% glycerol (*v*/*v*). WXD234 were cultured until the OD_600_ value ranged from 0.3–0.4 and were collected by centrifugation and washed 2× with 0.5 M saccharose, 0.5 mM KH_2_PO_4_, and 0.5 mM MgCl_2_. Each strain was suspended in its washing buffer (1:50), and the recombinant plasmid pNZ8148-Hla was introduced immediately into competent *Lactobacillus* by electroporation. Clones with the insertion of the target gene were screened by identifying antibiotic-resistant *Lactobacillus* by enzyme restriction, PCR, and sequencing. Positive clones of SXJ29 (pNZ8148-Hla) and WXD234 (pNZ8148-Hla) were frozen (−80 °C) in MRS containing 15% glycerol.

Hla_H35L_ expression in SXJ29 and WXD234 was assayed by Western blotting and confocal laser scanning microscopy (CLSM). SXJ29 (pNZ8148-Hla), SXJ29 (pNZ8148), WXD234 (pNZ8148-Hla), and WXD234 (pNZ8148) were cultured in MRS at 37 °C until the OD_600_ value ranged 0.6–0.8, induced with 5 ng/mL nisin (Sigma-Aldrich; St. Louis, MO, USA), and cultured overnight. Cells were washed 2× with 0.01 M phosphate-buffered saline (PBS; Coolaber Science & Technology Co.; Beijing, China), lysed in PBS containing 10 mg/mL lysozyme (Coolaber) for 30 min at 37 °C, sonicated at 150 W for 10 min, and boiled for 10 min. Lysates were subjected to 12% SDS-PAGE followed by Western blotting analysis using mouse anti-Hla_H35L_ mAb (GenScript Biotech) as primary the antibody and HRP-conjugated goat anti-mouse IgG (Proteintech; Rosemont, IL, USA) as the secondary antibody. The location of Hla_H35L_ was assayed through immunofluorescence assay as follows: the cells were collected, washed 3× with PBS, blocked for 1 h with 3% BSA in PBS, stained with anti-Hla_H35L_ mAb (GenScript Biotech) for 1 h, incubated with Cy3-conjugated goat anti-mouse IgG (Sangon, Biotech; Beijing, China) for 1 h, and incubated again with antigen-free strains SXJ29 (pNZ8148) and WXD234 (pNZ8148) as a negative control. The red fluorescent signal was acquired using a 550 nm excitation laser line and was detected at 560 to 600 nm, and photographs were taken with a Nikon camera.

We measured the physiological indexes of SXJ29 (pNZ8148-Hla) and WXD234 (pNZ8148-Hla), i.e., including growth curves, growth states at various temperatures (16, 23, 30, 37, 42, 49, 56 °C), pH values (2.0, 3.0, 4.0, 5.0, 6.0, 7.0, 8.0, 9.0), NaCl concentrations (0, 1, 3, 5, 7, 9, 11%), acid resistance, and bile tolerance [30]. The hereditary stability of recombinant strain detection was conducted according to a published protocol [31]. In brief, the recombinant strains serially transferred the cultures after 24 h of incubation in MRS medium containing chloramphenicol at 37 °C (20 generations), and the plasmid was extracted to confirm the presence of recombinant plasmids in each strain.

### 2.4. Colonization and Persistence of Recombinant Lactobacillus in the Intestine

To monitor the colonization and persistence of recombinant *Lactobacillus* in vivo, EGFP-expressing *Lactobacillus* SXJ29 (pNZ8148-EGFP) and WXD234 (pNZ8148-EGFP) were constructed the same as described above. Mice were divided into SXJ29 (pNZ8148-EGFP) (*n* = 15), WXD234 (pNZ8148-EGFP) (*n* = 15), and control groups (*n* = 3). All of the mice were orally inoculated with 2 × 10^9^ colony-forming units (CFUs) of bacteria or PBS, and intragastric administration was performed for three consecutive days. Prior to intragastric administration, recombinant *Lactobacillus* were cultured in MRS until the OD_600_ value ranged from 0.6–0.8, induced with 5 ng/mL nisin for 6 h, washed with PBS, and concentration adjusted to 10^10^ CFU/mL. The intestines (duodenum, jejunum, ileum, cecum, and colon) were collected at 6, 12, 24, 48, and 72 h after the last intragastric administration. To assess the mucosal-associated bacteria, the entire length of the intestine was flushed with ice-cold sterile PBS that removed and collected the luminal contents and loosely adherent bacteria. Then, the intestinal tissues were homogenized using a tissue homogenizer (Analytikjena; Yena, Germany) in 5 mL of PBS containing 0.1% Tween-20 (Coolaber) (PBST). Samples from the luminal contents and tissue homogenate were serially diluted in PBS and were cultured on MRS plates containing chloramphenicol at 37 °C for 48 h, and the bacterial colonies were counted using a Scan 300 automated colony counter (Interscience; Saint Nom la Bretêche, France). The intestinal contents of each group at 6 h were mounted on slides for direct observation with fluorescence microscopy (TI-DH inverted fluorescence Microscopy, Nikon) equipped with a GFP filter set (excitation 470 nm; emission 505 nm to 530 nm). Photographs were taken with a Nikon camera.

### 2.5. Vaccination

The preparation of the bacteria prior to intragastric administration was same as described above. All of the mice were orally inoculated with 2 × 10^9^ CFUs of bacteria or PBS. The immunization protocol was conducted over three consecutive days: days 1, 2, and 3. Booster immunization was administered on days 14, 15, and 16, and a second booster was given on days 28, 29, and 30.

### 2.6. Determination of IgG and IgA Levels by ELISA

Levels of Hla_H35L_-specific IgG in serum and IgA in the intestinal mucus were determined by enzyme-linked immunosorbent assay (ELISA). Sera and intestinal mucus were collected from three mice that were selected randomly from the vaccinated mice on days 1, 7, 14, 21, 28, 35, and 42. Serum samples were collected from the retroorbital plexus, kept at 37 °C for 2 h for clotting, centrifuged, and stored at −20 °C until testing. The intestinal mucus (each ~0.2 g) was separated, suspended with 500 μL HEPES buffer (Thermo Fisher Scientifics; Waltham, MA, USA), and centrifuged (3000× *g*, 15 min). Supernatant was stored at −20 °C until it was assayed. Next, 96-well MICROLON ELISA plates were coated with 0.5 μg/mL purified Hla_H35L_ protein (obtained from the *E. coli* expression system constructed by our previous work) overnight at 4 °C, washed 3× with PBS containing 0.05% Tween-20 (PBST), blocked with 5% BSA for 2 h at 37 °C, and washed 3× with PBST. Samples (serum at 1:50 dilution; intestinal mucus at 1:10 dilution) were added and incubated for 2 h at 37 °C, washed 5x with PBST, added with HRP-conjugated goat anti-mouse IgG or IgA (Proteintech) for 2 h at 37 °C, washed 5× with PBST. TMB single-component substrate solution (Solarbio; Beijing, China) was then employed to develop the color, and absorbance was measured as OD_450_.

### 2.7. T Cell Proliferation Assay

Spleens and Peyer’s patches were removed from three mice in each group on day 35, and we established single-cell suspensions, as described previously [32]. Red blood cells were lysed in hypotonic buffers, and the suspensions were cultivated in 75 cm^2^ flasks in a 5% CO_2_ incubator for the following T-lymphocyte proliferation assay. In brief, a 100 μL cell suspension (~10^5^ cells/mL) was incubated in a 96-well plate, which was stimulated by purified Hla_H35L_ protein (final concentration 20 μg/mL) (positive control: 10 μg/mL concanavalin A (ConA); negative control: RPMI 1640 medium) and was cultured for 72 h at 37 °C in a 5% CO_2_ incubator. T-lymphocyte proliferation was determined by Cell Titer 96 AQueous One Solution Cell Proliferation Assay (MTS) (Promega Corp.; Madison, WI, USA), with a determined absorbance of OD_490_. The stimulation index (SI) was calculated as the OD value of the stimulated cells divided by the OD value of the unstimulated control cells [33].

### 2.8. Cytokine Level Assay

Intestinal tissues were collected from three mice that had been randomly selected from the vaccinated mice on day 35, suspended with 500 μL 0.1% PBST, and homogenized by a tissue homogenizer. Cytokine interleukin 2 (IL-2), IL-4, IL-10, IL-17 and interferon γ (IFN-γ) in the supernatant were assayed by an ELISA kit (R & D Systems; Minneapolis, MN, USA). Methods were essentially the same as for antibody detection.

### 2.9. Mouse Model of S. aureus-Induced Pulmonary Infection

Vaccinated mice (female WT, TCR γ/δ-deficient and IL-17A-deficient C57BL/6) were anesthetized by an intraperitoneal (i.p.) injection of ketamine–xylazine (KX) and were intranasally inoculated with *S. aureus* USA300 at concentration 5 × 10^9^ CFUs/40 μL/mouse to initiate pulmonary infection on day 36. Survival and health status were monitored and recorded during a 72-h period following the challenge onset for the mice in each group (*n* = 10). For bacteriological and histological analyses, vaccinated mice were inoculated with *S. aureus* USA300 at concentration 5 × 10^8^ CFUs/40 μL/mouse on day 36 (*n* = 10). Following the challenge, the right lungs were removed at 48 h by thoracotomy, weighed, and homogenized. Serial dilutions of 100 μL tissue homogenate were placed on mannitol salt agar (MSA) plates (HuanKai Microbial Sci. & Tech. Co.; Guangdong, China), incubated overnight at 37 °C, and the bacterial colonies were counted using a Scan 300 automated colony counter. The left lungs were fixed, paraffin-embedded, sectioned (4–6 μm), stained with H&E, and evaluated by means of light microscopy (Olympus; Shinjuku, Japan). A total of three fields were examined, and the inflammation index was calculated as (number of inflammatory cells in visual field/total number of cells in field) × 100%.

### 2.10. Mouse Model of S. aureus-Induced Skin Infection

The vaccinated mice (female WT and TCR γ/δ-deficient C57BL/6) were anesthetized, inoculated by dorsal s.c. injection with 5 × 10^8^ CFUs/50 μL/mouse of *S. aureus* USA300, and monitored daily for 28 days for mass and abscess formation (*n* = 6). The sizes of the abscesses and the associated overlying dermonecrotic lesions were determined by a standard equation: Area (A) = (π/2) × length × width. For counting of *S. aureus* CFUs in skin abscess lesions, the animals were euthanized 1 day after inoculation, the abscesses were removed and homogenized in PBS, and the CFUs were counted by plating serially diluted samples on tryptic soy agar at 37 °C and by allowing them to grow for 24 h (*n* = 3). The skin tissues were fixed, paraffin-embedded, sectioned (4–6 μm), and stained with H & E (*n* = 3).

### 2.11. Statistical Analysis

Data were analyzed using the software program GraphPad Prism 8 (San Diego, CA, USA) and were presented as the mean ± SD from the three replicates for each experiment repeated 3×. Kaplan–Meier survival analysis was performed, and Log-rank (Mantel–Cox) tests were used to make the comparisons between the two groups. Other data were statistically analyzed by a *t*-test. Differences with *p* < 0.05 and *p* < 0.01 were considered to be significant and highly significant, respectively.

## 3. Results

### 3.1. Cell Surface Display System for Hla_H35L_ Expression in Transformed Lactobacillus

We constructed Hla_H35L_ cell surface display *Lactobacillus* using pNZ8148 and a strategy for the construction of pNZ8148-Hla, which are shown schematically in Figure 1a. The recombinant plasmid pNZ8148-Hla was constructed successfully (Figure 1d) and was introduced into SXJ29 and WXD234 by electroporation. SXJ29 (pNZ8148-Hla) and WXD234 (pNZ8148-Hla) lysates were analyzed by Western blotting. Specific signals were detected at molecular mass 40.3 kDa (Figure 1b), which is consistent with Hla_H35L_. Thus, the *S. aureus* antigen gene *hla_H35L_* was expressed successfully in SXJ29 and WXD234. The location of Hla_H35L_ was analyzed by CLSM, with SXJ29 (pNZ8148) and WXD234 (pNZ8148) as negative controls. Visible red fluorescence under green light excitation was observed for SXJ29 (pNZ8148-Hla) and WXD234 (pNZ8148-Hla) but not for the negative control strains (Figure 1f). This CLSM finding indicates that Hla_H35L_ was successfully expressed on the cell surface of lactobacilli.

**Figure 2:**
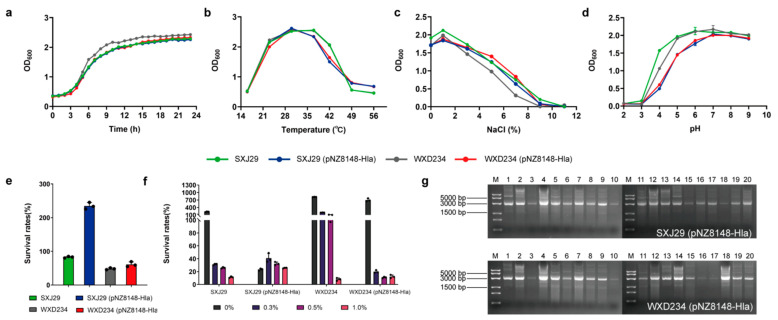
The physiological characteristics of recombinant *Lactobacillus* SXJ29 (pNZ8148-Hla), WXD234 (pNZ8148-Hla), and the wild strains SXJ29 and WXD234. (**a**) The growth curves of strains for 23 h were determined (*n* = 3). (**b**) Growth states of the strains under different temperatures (16, 23, 30, 37, 42, 49, 56 °C) (*n* = 3). (**c**) Growth states of the strains at different pH values (2.0, 3.0, 4.0, 5.0, 6.0, 7.0, 8.0, 9.0) (*n* = 3). (**d**) Growth states of the strains at the different NaCl contents (0%, 1%, 3%, 5%, 7%, 9%, 11%) (*n* = 3). Data are shown as mean ± SD for each group. (**e**) The acid tolerance of the strains was assessed in terms of viable colony counts after incubation with artificial gastric juice (NaCl 0.2% and pepsin 0.35% were dissolved in distilled water, and the pH was adjusted to 3.0, and filter sterilized with 0.22 μm micro) at 37 °C for 3 h (*n* = 3). (**f**) The bile tolerance of the strains was assessed in terms of viable colony counts after incubation with artificial bile salt intestinal juice (MRS broth with 0.68% KH_2_PO_4_ and 1% trypsin, and the content of the bile salt was 0%, 0.3%, 0.5%, and 1.0%) at 37 °C for 0 and 24 h (*n* = 3). (**g**) The plasmid pNZ8148-Hla have genetic stability in both SXJ29 (pNZ8148-Hla) and WXD234 (pNZ8148-Hla) and can stabilize the heredity for at least 20 d.

Recombinant Lactobacillus and WT strains did not differ notably in terms of growth curves (Figure 2a), growth status at various temperatures (Figure 2b), pH value (Figure 2c), or NaCl content (Figure 2d). Recombinant Lactobacillus retained acid (Figure 2e) and bile salt resistance properties (Figure 2f), and plasmid pNZ8148-Hla showed stabilized inheritance of at least 20 d (Figure 2g).

### 3.2. Recombinant Lactobacillus Colonize and Express Heterologous Protein Stably in the Intestine

**Figure 3:**
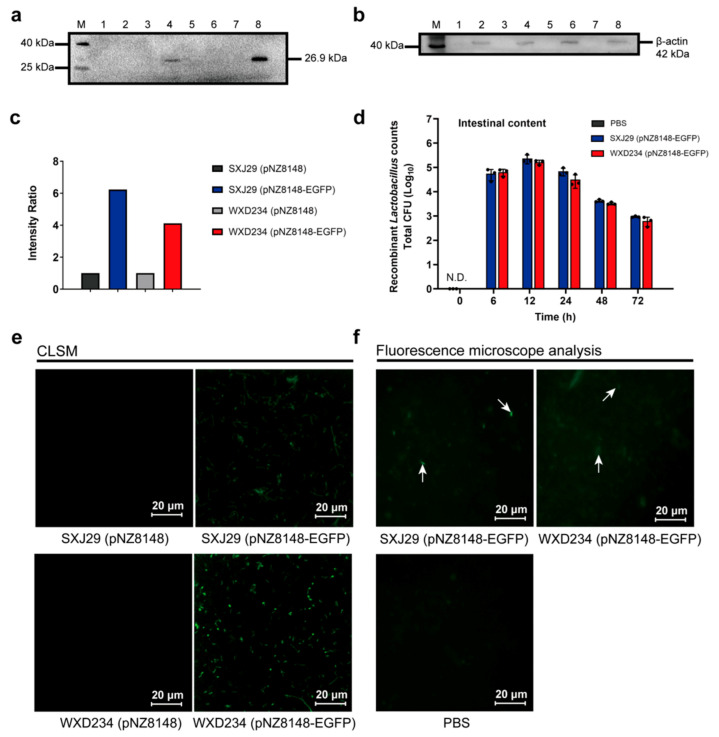
Recombinant *Lactobacillus* can persist for at least 72 h and can stably express heterologous protein in the intestine. Recombinant *Lactobacillus* SXJ29 (pNZ8148-EGFP) and WXD234 (pNZ8148-EGFP) were constructed, and EGFP expression on recombinant SXJ29/WXD234 (pNZ8148-EGFP) subjected to nisin induction was measured by Western blotting and CLSM. Culture supernatants and cell extracts were prepared and analyzed by Western blotting using anti-GFP mAb (**a**) or anti-β-actin mAb (**b**). Left: molecular masses of pre-stained marker proteins. Protein marker (Lane M). Culture supernatants of SXJ29 (pNZ8148) (Lane 1), SXJ29 (pNZ8148-EGFP) (Lane 3), WXD234 (pNZ8148) (Lane 5), WXD234 (pNZ8148-EGFP) (Lane 7); cell extracts of SXJ29 (pNZ8148) (Lane 2), SXJ (pNZ8148-EGFP) (Lane 4), WXD234 (pNZ8148) (Lane 6), and WXD234 (pNZ8148-EGFP) (Lane 8). (**c**) Intensity ratio of EGFP expression in SXJ29 and WXD234. (**e**) CLSM detection of EGFP expression on SXJ29/WXD234 (pNZ8148-EGFP). (**d**) Mice were orally administered SXJ29 (pNZ8148-EGFP), WXD234 (pNZ8148-EGFP) or PBS, and recombinant *Lactobacillus* colonization was monitored for 72 h (*n* = 3). (**f**) Fluorescent detection of SXJ29/WXD234 (pNZ8148-EGFP) in the intestine 6 h after the oral administration of recombinant *Lactobacillus* under fluorescence microscopy.

To monitor the colonization and persistence of recombinant *Lactobacillus* in vivo, recombinant *Lactobacillus* SXJ29 (pNZ8148-EGFP) and WXD234 (pNZ8148-EGFP) were constructed. EGFP can be stably expressed in recombinant *Lactobacillus* SXJ29 (pNZ8148-EGFP) and WXD234 (pNZ8148-EGFP) in vitro by Western blotting and CLSM analysis (Figure 3a,e).

The mouse experimental results showed that the geometric means of the total SXJ29/WXD234 (pNZ8148-EGFP) CFUs in the intestinal contents were analyzed (Figure 3d) at 6, 12, 24, 48, and 72 h after the last intragastric administration with transformant. The data suggested that recombinant lactobacilli were able to colonize and persist, albeit at a reduced level, in the gastrointestinal tract, even at 72 h after post-administration. There was no significant difference in the amount of colonization between the SXJ29 (pNZ8148-EGFP) and WXD234 (pNZ8148-EGFP) strains. Using fluorescence microscopy, it became apparent that fluorescent lactobacilli was contained in the intestinal contents of the mice 6 h post-administration (Figure 3f). These results indicate that recombinant *Lactobacillus* can persist in the intestine for at least 72 h and that heterologous protein can be stably expressed in the intestine.

### 3.3. Recombinant Lactobacillus Elicited Intestinal Mucus IgA and Serum IgG-Mediated Immune Responses

**Figure 4:**
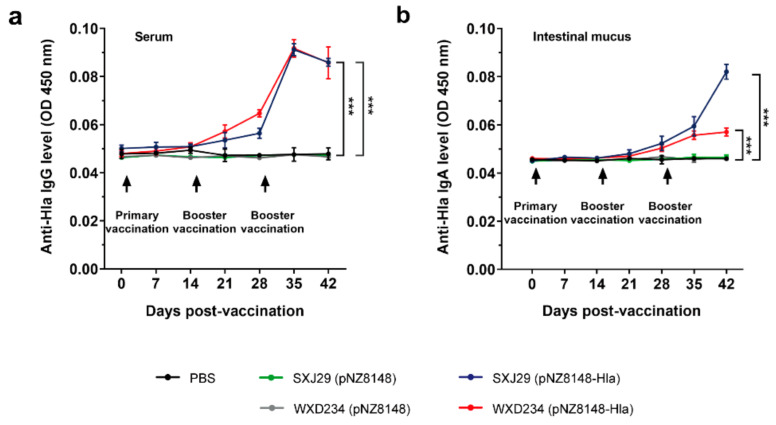
Recombinant *Lactobacillus* elicited IgG- and IgA-mediated immune responses. Mice were vaccinated with SXJ29 (pNZ8148-Hla), WXD234 (pNZ8148-Hla), or PBS; samples were collected at 7-day intervals as indicated, and the IgG and IgA levels were measured. (**a**) Mean of Hla_H35L_-specific IgG levels in serum (*n* = 3). (**b**) Mean of Hla_H35L_-specific IgA levels in intestinal mucus (*n* = 3). Data are shown as mean ± SD for each group. *** *p* < 0.001 for comparison with PBS group.

To examine the activation of humoral and mucosal immunity by recombinant SXJ29 (pNZ8148-Hla) and WXD234 (pNZ8148-Hla) from an antibody perspective, we measured serum IgG and intestinal mucus IgA levels. Hla_H35L_-specific IgG was detected in sera from all of the vaccinated mice on days 0, 7, 14, 21, 28, 35, and 42 (Figure 4a). On days 28, 35, and 42, levels differed significantly, and on day 35, the levels were the highest. Levels of Hla_H35L_-specific IgA that had been assayed in gastroenteric mucus showed a steady increase and peaked on day 42 in all of the vaccinated mice (Figure 4b). Taken together, these findings indicate that SXJ29 (pNZ8148-Hla) and WXD234 (pNZ8148-Hla) elicited IgG- and IgA-mediated immune responses.

### 3.4. Recombinant Lactobacillus Enhanced Lymphocyte Proliferation in Peyer’s Patches

**Figure 5:**
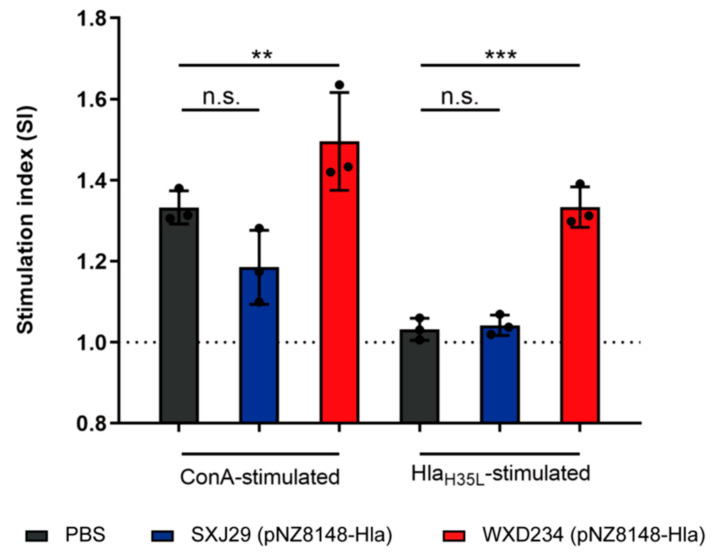
Recombinant *Lactobacillus* enhanced lymphocyte proliferation in Peyer’s patches. Mice were vaccinated with SXJ29 (pNZ8148-Hla), WXD234 (pNZ8148-Hla), or PBS. On day 35, T-lymphocytes from Peyer’s patches were prepared, restimulated with Hla_H35L_ or Con A protein, and measured by MTS assay (*n* = 3). Results are shown as the stimulation index (SI). Data are shown as mean ± SD for each group. ** *p* < 0.01; *** *p* < 0.001; n.s., not significant for comparison with PBS group.

T cell suspensions from the spleen and Peyer’s patches were prepared from vaccinated mice on day 35 to further evaluate the effects of recombinant *Lactobacillus* SXJ29 (pNZ8148-Hla) and WXD234 (pNZ8148-Hla) on cell-mediated immunity, and a MTS assay of the T-lymphocytes proliferation was performed. For both Hla_H35L_- and Con A-stimulated Peyer’s patch lymphocytes, a significant enhancement in proliferation was observed for the WXD234 (pNZ8148-Hla) group, but feeble proliferation was observed for the SXJ29 (pNZ8148-Hla) group (Figure 5). The spleen T-lymphocytes showed no notable proliferation (data not shown). Thus, WXD234 (pNZ8148-Hla) apparently stimulated cell-mediated immune responses in the gut-associated lymphoid tissue (GALT).

### 3.5. Recombinant Lactobacillus Enhanced Production of IL-2, IL-4, and IL-17 in Mesenteric Lymphatic Tissue

**Figure 6:**
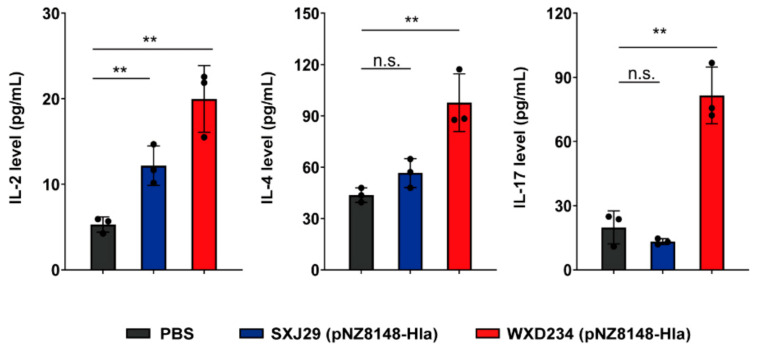
WXD234 (pNZ8148-Hla) vaccination induced IL-2, IL-4 and IL-17 production of in mesenteric lymphatic tissue. Vaccinated mice were sacrificed on day 35, and the intestinal tissue was prepared. IL-2, IL-4, and IL-17 levels, determined by ELISA in a supernatant of intestinal tissue homogenate were analyzed (*n* = 3). Data are shown as mean ± SD for each group. ** *p* < 0.01; n.s., not significant for comparison with PBS group.

T-helper (Th) cells mainly differentiate into Th1, Th2, and Th17 subsets [34] and play essential roles in promoting immune responses following bacterial infection or immunization [35,36,37]. Cytokines secreted by the T cells can be classified into Th1-related (IFN-γ, IL-2), Th2-related (IL-4, IL-10), and Th17-related (IL-17) cytokines [38,39]. We prepared a supernatant of the intestinal tissue homogenate in order to investigate the effects of our recombinant *Lactobacillus* on the production of IL-2, IL-4, IL-10, IL-17, and IFN-γ by mesenteric lymphocytes. The IL-2, IL-4, and IL-17 levels were significantly higher in the WXD234 (pNZ8148-Hla)-vaccinated group than in the PBS group (Figure 6). The IL-2 and IL-4 levels were higher in the SXJ29 (pNZ8148-Hla)-vaccinated group than in the PBS group, but the difference was not significant. These findings indicate that WXD234 (pNZ8148-Hla) is capable of inducing Th1, Th2, and Th17 cell-mediated immune responses.

### 3.6. Recombinant Lactobacillus Effectively Protected Mice against S. aureus-Induced Pulmonary Infection

**Figure 7:**
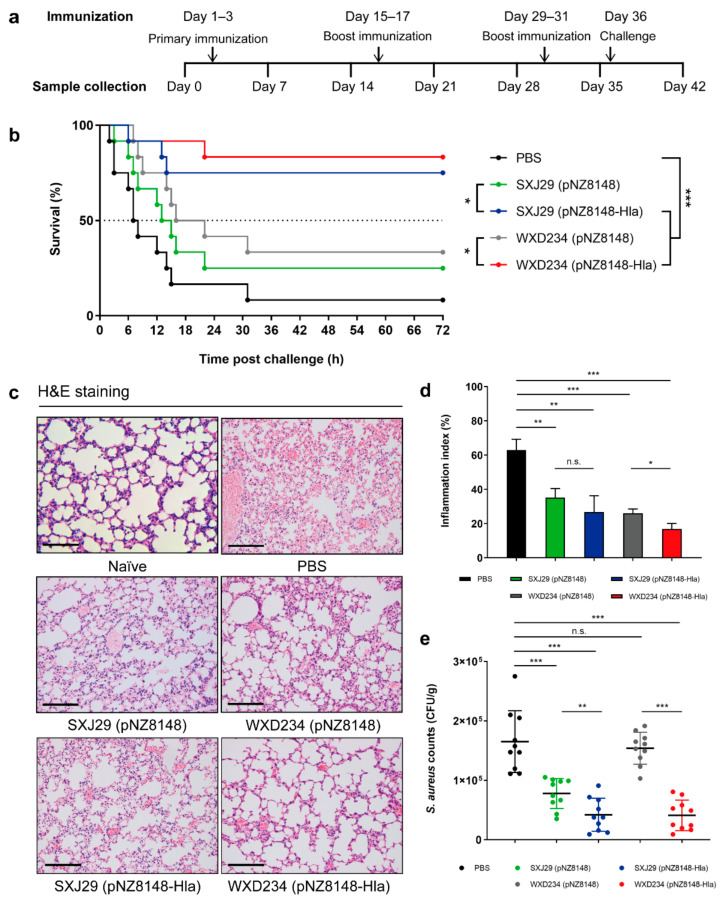
Recombinant *Lactobacillus* effectively improved the protection against *S. aureus*-induced pulmonary infection. (**a**) Immunization strategy and schedule for the collection of serum, feces, intestinal tissue, and intestinal mucus samples. (**b**) Vaccinated mice were inoculated with 40 μL bacterial slurry (5 × 10^9^ CFUs of *S. aureus* USA300) via the left nostril on day 36 and were held upright for 1 min. Survival rates were monitored for 72 h (*n* = 12). (**c**) Vaccinated mice were inoculated with 40 μL bacterial slurry (lower dose: 5 × 10^8^ CFUs of *S. aureus* USA300), as above. Histopathological evaluation of lung sections by light microscopy (H&E staining; magnification 200×; bar: 100 μm). (**d**) Inflammation index corresponding with histopathological evaluation (*n* = 10). (**e**) For the lower-dose (5 × 10^8^ CFUs) group, the numbers of bacteria in lungs were counted (*n* = 10). * *p* ˂ 0.05; ** *p* < 0.01; *** *p* < 0.001; n.s., not significant.

To evaluate the effect of recombinant *Lactobacillus* on resistance to *S. aureus* infection, we constructed a *S. aureus*-induced pulmonary infection model. After intranasal challenging, survival rates were high for the WXD234 (pNZ8148-Hla) (83%; 10/12) and SXJ29 (pNZ8148-Hla) (75%; 9/12) groups but were much lower for the PBS (8%; 1/12), WXD234 (pNZ8148) (33%; 4/12), and SXJ29 (pNZ8148) (25%; 3/12) antigen-free groups (Figure 7b). Moreover, the degree of *S. aureus* colonization in the lung was much lower for the SXJ29 (pNZ8148-Hla) and WXD234 (pNZ8148-Hla) groups than for the PBS group (Figure 7e). Meanwhile, the SXJ29 (pNZ8148-Hla)- or WXD234 (pNZ8148-Hla)-vaccinated mice only exhibited mild inflammatory reactions, whereas severe histopathological damage, including fragmentation of alveolar walls, infiltration of lymphocytes, and a large amount of erythrocyte exudation, were observed in PBS-treated mice (Figure 7c). Moreover, the lung histopathology scores (inflammation index), as defined by the percentage leukocyte infiltration cells, were significantly reduced in the SXJ29 (pNZ8148-Hla) and WXD234 (pNZ8148-Hla) groups compared to in the PBS group (Figure 7d). Although the antigen-free groups SXJ29 (pNZ8148) and WXD234 (pNZ8148) had a certain degree of protective ability, the recombinant bacteria containing Hla_H35L_ could further improve the protective effect. Both the *Lactobacillus* vector and Hla_H35L_ played important roles in this protective effect. These data therefore demonstrate the therapeutic effects of recombinant *Lactobacillus* in improving lung physiology and histopathology in vivo and support the potential use of SXJ29 (pNZ8148-Hla) and (particularly) WXD234 (pNZ8148-Hla) for protection against *S. aureus*-induced pulmonary infection.

### 3.7. Recombinant Lactobacillus Promoted Resistance to S. aureus-Induced Skin Infection

**Figure 8:**
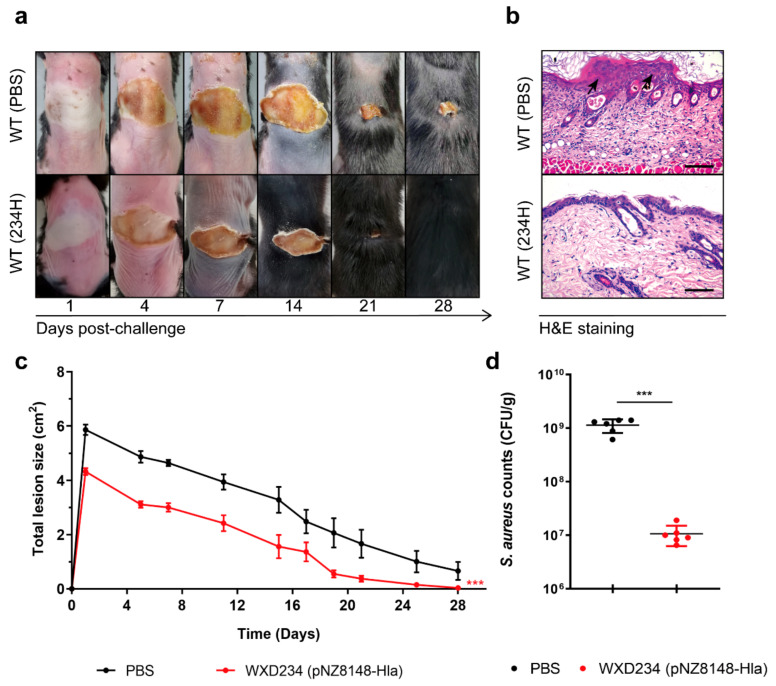
Recombinant *Lactobacillus* WXD234 (pNZ8148-Hla) promoted resistance to *S. aureus*-induced skin infection. (**a**) WT C57BL/6 mice were vaccinated with WXD234 (pNZ8148-Hla) or PBS and then challenged with *S. aureus* USA300 by the dorsal s.c. injection of 5 × 10^8^ CFUs/50 μL/mouse on day 36, and the lesion sizes were monitored for 28 days (*n* = 6). Representative photographs of skin lesions are shown. (**c**) Total lesion sizes (cm^2^) ± SEM (*n* = 6) are shown. *** *p* < 0.001 for comparison with the PBS group. (**b**) Histopathological evaluation of skin infection (H & E staining; magnification 200×; bar: 200 μm; *n* = 3). (**d**) Number of bacteria in lesions were counted (*n* = 3) at 24 h post infection. Data are shown as mean ± SD for each group. *** *p* < 0.001 for comparison with PBS group.

The skin, the body’s largest mucosa organ, is home to a diverse and complex variety of innate and adaptive immune functions that protect against pathogenic invasion. To evaluate the effect of recombinant *Lactobacillus* WXD234 (pNZ8148-Hla) on resistance to *S. aureus* infection, we then constructed a *S. aureus*-induced skin infection model. Vaccinated mice were intradermally inoculated with *S. aureus* USA300 (5 × 10^8^ CFUs/50 μL/mouse) and developed visible skin lesions. The skin lesions of the WXD234 (pNZ8148-Hla)-vaccinated mice reached a maximal size of 4.32 ± 0.13 cm^2^ by day 2 and healed by day 28 (Figure 8a,c). The non-vaccinated mice developed much larger lesions (5.87 ± 0.19 cm^2^ by day 2), which were still not completely healed by day 28 (Figure 8a,c). Histological evaluation (H&E staining) of the skin at 24 h revealed large neutrophilic abscesses in the WXD234 (pNZ8148-Hla)-vaccinated mice (Figure 8b). The numbers of *S. aureus* CFUs in the infection site at 24 h were ~100-fold higher in the non-vaccinated mice than in the WXD234 (pNZ8148-Hla)-vaccinated mice (Figure 8d). Thus, recombinant Lactobacillus WXD234 (pNZ8148-Hla) promoted resistance to *S. aureus*-induced skin infection.

### 3.8. Recombinant Lactobacillus Lost Superior Protection Post Vaccination in TCR γ/δ-Deficient or IL-17A-Deficient Mice

**Figure 9:**
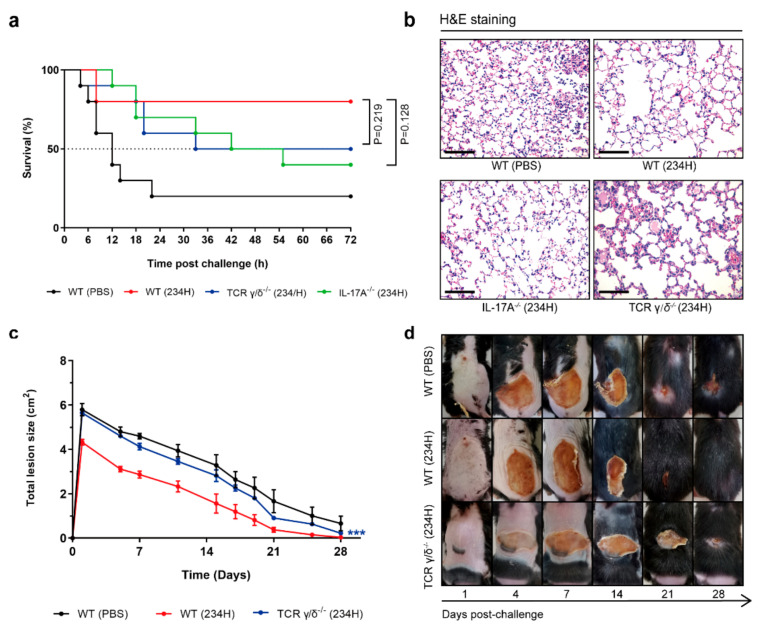
Protection offered by WXD234 (pNZ8148-Hla) vaccination decreased when γδ T cell or IL-17 absent. (**a**) WT, IL-17A^−/−^, and TCR γ/δ^−/−^ C57BL/6 mice vaccinated with WXD234 (pNZ8148-Hla) were inoculated with 60 μL bacterial slurry (2.2 × 10^10^ CFUs *S. aureus* USA300), as described above. Survival rates were monitored for 72 h (*n* = 10). (**b**) Vaccinated mice were inoculated with 50 μL bacterial slurry (lower dose: 5 × 10^8^ CFUs of *S. aureus* USA300), as described above. Histopathological evaluation of lung sections by light microscopy (H & E staining; magnification 200×; bar: 100 μm). (**c**) Total lesion size (cm^2^) ± SEM (*n* = 6) are shown. *** *p* < 0.001 for comparison between TCR γ/δ^−/−^ (234H) and WT (234H) groups. (**d**) WT and TCR γ/δ^−/−^ C57BL/6 mice vaccinated with WXD234 (pNZ8148-Hla) were challenged with *S. aureus* USA300 by dorsal s.c. injection of 5 × 10^8^ CFUs/50 μL/mouse on day 36, and lesion sizes were monitored for 28 days (*n* = 6). Representative photographs of skin lesions are shown.

γδ T cells are an important subset of “unconventional” T-lymphocytes that are present in epithelial tissues, including in the gastrointestinal tract, skin epidermis, and reproductive tract [40]. γδ T cells function as a bridge between innate and adaptive immune responses and therefore have essential roles in mucosal immunity [41]. γδ T cells evidently play a key role in the immune response to *S. aureus* skin infections [42]. The contribution of γδ T cells to *S. aureus*-induced pulmonary and skin infection was evaluated by vaccinating the WT and TCR γ/δ-deficient mice with WXD234 (pNZ8148-Hla) in our model system. A murine model of a pulmonary infection model was established, and survival rates were calculated on day 36. The survival rate was significantly higher for the WT (234H) group (80%; 8/10) than for the WT (PBS) group (20%; 2/10) or the TCR γ/δ^−/−^ (234H) group (50%; 5/10) (Figure 9a). The histological evaluation of these groups in our *S. aureus*-induced pulmonary infection model was based on H&E staining and a light microscopic examination of the lung sections. The WT (234H) group had clear alveolar airspaces, continuous thin alveolar walls and capillary vessels, clearly delineated extra-alveolar vessels with no evident patent lymphatics in adventitia, and a small number of neutrophils, which were confined to the septal network (Figure 9b). In contrast, the WT (PBS) and TCR γ/δ^−/−^ (234H) groups displayed interstitial edema, neutrophil infiltration, and thickened alveolar walls. Meanwhile, the contribution of γδ T cells to *S. aureus*-induced skin infection was evaluated by vaccinating WT and TCR γ/δ-deficient mice with WXD234 (pNZ8148-Hla) and by measuring the lesion size in our model system. The WXD234 (pNZ8148-Hla)-vaccinated mice developed lower skin lesions. In contrast, vaccinated TCR γ/δ^−/−^ mice developed lesions that were ~1.5 to 2-fold larger and that did not heal completely by day 21; however, this is still lower than for the non-vaccinated mice (Figure 9c,d). Taken together, these results indicate that recombinant *Lactobacillus* WXD234 (pNZ8148-Hla) lost superior protection post vaccination in the TCR γ/δ-deficient mice in both the *S. aureus*-induced pulmonary infection and skin infection groups.

The increased IL-17 level produced by WXD234 (pNZ8148-Hla) vaccination was of particular interest. IL-17 plays a key role in host defense against *S. aureus* infection, and immunomodulatory therapies or vaccines that enhance IL-17 response have strong potential in this regard [43]. We investigated roles of IL-17 by evaluating the protective effect of WXD234 (pNZ8148-Hla) in WT and IL-17A^−/−^ C57BL/6 mice. Survival rates were significantly lower for the IL-17A^−/−^ (234H) group (40%; 4/10) than for the WT (234H) group (80%; 8/10) (Figure 9a). The histological evaluation of IL-17A^−/−^ (234H) group displayed significant histopathological damage (Figure 9b). These findings indicate that WXD234 (pNZ8148-Hla) provided effective protection against *S. aureus*-induced pulmonary infection, and this protection decreased when IL-17 was absent.

**Table 1:** Bacterial strains and plasmids used in this study.

Strain or Plasmid	Description	Source or Reference
Strains		
SXJ29	WT *Lactobacillus kefiri*	Isolated from dairy products
WXD234	WT *Lactobacillus plantarum*	Isolated from dairy products
SXJ29 (pNZ8148)	Derivative of SXJ29 carrying pNZ8148	This study
WXD234 (pNZ8148)	Derivative of WXD234 carrying pNZ8148	This study
SXJ29 (pNZ8148-Hla)	Derivative of SXJ29 carrying pNZ8148-Hla	This study
WXD234 (pNZ8148-Hla)	Derivative of WXD234 carrying pNZ8148-Hla	This study
SXJ29 (pNZ8148-EGFP)	Derivative of SXJ29 carrying pNZ8148-EGFP	This study
WXD234 (pNZ8148-EGFP)	Derivative of WXD234 carrying pNZ8148-EGFP	This study
*S. aureus* USA300	*S. aureus* USA300	American Type Culture Collection (Manassas, VA, USA)
Plasmids		
pUC57-Hla	Amp^r^; pUC57::_SP_*slpA*::his-tag::*hla_H35L_*	GenScript Bio Co.
pNZ8148	Cm^r^; *L. lactis* expression vector; P_nisA_ promoter	Nanjing ZFdows Bio Co. [44]
pNZ8148-Hla	Cm^r^; pNZ8148::_SP_*slpA*::his-tag::*hla_H35L_*	This study
pNZ8148-EGFP	Cm^r^; pNZ8148::*egfp*	This study

## 4. Discussion

Numerous scientific institutions and research groups have attempted to develop effective vaccines against *S. aureus* during the past two decades, but so far, these attempts have had limited success [6]. Such attempts face major challenges that have been discussed extensively in several review articles [6,7]. At present, research based on *Lactococcus* accounts for the majority of the research that has been conducted in the development of a lactic acid bacteria (LAB) delivery system because *Lactococcus* was the first species of LAB to have its genome fully sequenced, which has allowed a better understanding of its physiological mechanisms and gene editing manipulation [45,46,47,48]. In recent years, lactobacilli, which have low innate immunogenicity and low modulating non-specific immunity, have been widely used to develop oral vaccines as delivery vehicles [49,50,51]. However, the possible use of lactobacilli as a delivery system for a *S. aureus* vaccine has not been investigated. For this purpose, we designed oral vaccines based on *L. kefiri* SXJ29 and *L. plantarum* WXD234, taking advantage of their adaptability to the gastrointestinal environment and their probiotic properties. Recombinant *Lactobacillus* WXD234 (pNZ8148-Hla) provides superior immunoprotective effects in mouse models of *S. aureus*-induced pulmonary and skin infections.

When *S. aureus* initially colonizes an infection site, it affects the host immune system by producing virulence factors [52]. α-hemolysins (Hla) plays key roles in the pathogenesis of *S. aureus*-induced infections, which kill many types of cells through formation of heptameric membrane pores [53,54]. We created Hla mutants to minimize toxic effects. Previous studies have shown that active immunization with non-toxic mutant α-hemolysins (Hla_H35L_) that are unable to form pores or passive immunization with Hla_H35L_-specific antisera improve the protection against *S. aureus*-induced pulmonary and skin infections [55,56]. Lactobacilli vehicles have strong potential as vaccine vectors because of their ability to function as both adjuvants and vectors, enhancing the effect of the exogenous antigen on the systemic immune system [57]. We selected Hla_H35L_ as an antigen and SXJ29 and WXD234 as recipient bacteria for the construction of an anti-*S. aureus* oral vaccine. The surface displayed proteins in *L. lactis* were considered more stable and had a higher bioactivity than their secreted counterparts [58,59]. Therefore, Hla_H35L_ was linked to a 90-bp fragment encoding a *L. brevis* S-layer signal peptide, thus inducing more efficient secretion and transportation of the target protein to the cell surface to facilitate Hla_H35L_ function [29,60].

The balance or ratio of Th1, Th2, and Th17 cells is important for a healthy immune response [61]. IFN-γ produced by CD8^+^ T cells and certain CD4^+^ T cells (especially Th1 cells) can enhance the activity of Th1 cells and can promote cellular immune function [62]. The Th2-type immune response is marked by the secretion of cytokine IL-4 and the induction of specific antibodies [63]; the Th17-type is marked by IL-17 and IgA [64]. Oral vaccination has been shown to elicit mucosal immune responses; in particular, effective intestinal mucosal immune response requires the production of both mucosal secretory IgA (sIgA) and serum IgG antibodies to inhibit colonization by pathogens and their further spread to systemic circulation [65,66]. Recombinant *Lactobacillus* WXD234 (pNZ8148-Hla) apparently induces robust mucosal immunity in the GALT, as evidenced by a significant increase in IgA (Figure 4a) and IL-17 (Figure 6) production and the strong proliferation of the T-lymphocytes derived from Peyer’s patches (Figure 5). However, WXD234 (pNZ8148-Hla) could not stimulate a systemic immune response effectively because of the feeble proliferation of the T-lymphocytes derived from the spleen (data not shown) and the low level of IgG (Figure 4a). Nevertheless, the recombinant *Lactobacillus* showed a high protective effect in subsequent mouse models of *S. aureus*-induced pulmonary and skin infection, which was attributed to a strong mucosal immune response.

The use of an animal model reflecting the characteristics of clinical *S. aureus* infection is an essential evaluation method for the preclinical studies of vaccine. The mouse pulmonary infection model was established to simulate the infection pathways of *S. aureus* and was used for the evaluation of the immunogenicity and the immunoprotective effect of the *S. aureus* oral vaccines. This provided considerable experimental reference for the development of a *S. aureus* vaccine. As a result, data on survival rates, bacterial colonization, and pathological analysis in the pulmonary infection model (Figure 7) clearly indicate stronger resistance to infection in the vaccinated groups relative to the antigen-free and PBS groups, e.g., the survival rate was 83% for the WXD234 group vs. 8% for the PBS group. However, antigen-free *Lactobacillus* still have a degree protection, which proved that both lactobacilli and Hla_H35L_ play important roles in this protective effect. It is a remarkable fact that the oral vaccine based on *L. plantarum* WXD234 is better than that based on *L. kefiri* SXJ29 in terms of survival rate, pulmonary inflammation, or bacteria burden. Both the vehicle bacteria and the antigen contribute to the protective efficacy provided by a bacterial vaccine. As adjuvants, *Lactobacillus* influence innate immune cells such as macrophage or DCs, which, in turn, promote antigen presentation and the production of protective antigen specific antibodies in response to vaccination [67]. Different species of *Lactobacillus*, even different strains, show diverse adjuvant properties against bacterial or viral infection. The major immunological benefit of lactobacilli is the modulation of the host immune responses via interacting with the GI mucosa [68]. Different *Lactobacillus* strains may activate different components of the immune response due to specific capacities to produce particular surface molecules and the secretion of various proteins and metabolites that influence the host cells [69,70,71]. In previous studies, we found that compared toSXJ29, depending on the full advantage of their own probiotic features, WXD234 has a good immunoprotective effect in mouse models of *S. aureus*-induced pulmonary infection. The superior protection of WXD234 (pNZ8148-Hla) may be related to its induction of higher levels of IL-17 production. The WXD234 mechanism exerts a superior immunomodulatory effect that is not precisely known; however, we still need further studies. In addition, the difference in the immunoprotective effects between the two strains may be caused by the different expression levels of the antigen, and expression levels of antigen need quantification. The skin, the body’s largest mucosa organ, is home to a diverse and complex variety of innate and adaptive immune functions that protect against pathogenic invasion. We evaluated the protective effect of WXD234 (pNZ8148-Hla), which has shown great immune protection in the above findings in a mouse model of a *S. aureus*-induced skin infection. WXD234 (pNZ8148-Hla) could significantly reduce the abscess size in the *S. aureus* skin infection model.

The levels of Th1-type (IL-2), Th2-type (IL-4), and Th17-type (IL-17) cytokines in the mesenteric lymphatic tissue were significantly higher for the WXD234-vaccinated group than for the PBS group (Figure 7), indicating the strong induction of the Th1-, Th2-, and Th17-cell responses by WXD234. There has been some controversy regarding the immunoprotective properties of *S. aureus* vaccines. Currently, the general consensus is that cell-mediated immunity (Th1/Th17) and neutrophil activation are essential for effective immunoprotection, while antibodies play supporting roles in the opsonization and/or neutralization of virulence factors [72,73,74]. Increased levels of cytokine IL-17 in the WXD234 group appears to be a crucial step in the potentiation of a mucosal immune response against foreign antigens. IL-17 is predominantly produced by Th17 cells [75] and plays a key role in a variety of immune and inflammatory responses by regulating the expression of inflammatory mediators such as other cytokines, chemokines, and adhesion molecules [76,77,78]. γδ T cells provide a link between innate and adaptive immunity and are mainly distributed in skin and mucosal tissues [78]. We conducted a preliminary investigation on the role of IL-17 and γδ T cells in the protective effect of WXD234 in WT, IL-17A^−/−^, and TCR γ/δ^−/−^ C57BL/6 mice. Of particular interest is the sharp reduction of are protective effect offered by WXD234 (pNZ8148-Hla) when γδ T cells or cytokine IL-17 are absent. Thus, γδ T cells or IL-17 may be involved in the protective effect of WXD234 (pNZ8148-Hla) against *S. aureus*-induced pulmonary or skin infections.

In summary, the genetically engineered *S. aureus* oral vaccines SXJ29 (pNZ8148-Hla) and WXD234 (pNZ8148-Hla) constructed in this study effectively induced immune responses and improved protection against *S. aureus*-induced pulmonary infection in mouse models. Moreover, WXD234 (pNZ8148-Hla) in particular significantly reduced the abscess size in the *S. aureus* skin infection model and is a promising candidate for future clinical application.

## 5. Conclusions

The oral delivery of novel recombinant *Lactobacillus* elicits high protection against *S. aureus* pulmonary and skin infections. Our findings provide a great potential for a delivery system based on lactobacilli and provide experimental data for the development of *S. aureus* mucosal vaccines.

## Data Availability

All data that this study is based upon are available from the corresponding author upon request.
